# 

**Published:** 1915-07-10

**Authors:** 


### CONTENTS.

PAGE

**Scottish Medical Service Emergency Committee** ... ... ... ... ... ... ... 301

**Suicide Made Easy** ... ... ... ... ... 302

**Hospital and Institutional News** ... ... ... 303

The King's Investiture: Notice—The Spirit Tax and the British Medical Association—The Ranelagh Red Cross Fête—St. Marylebone Guardians and the Coal Supply—The Hospital Egg—A Suggested Economy in the Egg Bill—Needlework and Linen Guilds at Cardiff—Geriatrics—The Peter Pan of Forty—A Royal Army Dental Corps?—"Places of Safety" under the Mental Deficiency Act—A Surcharge for Assistance at Operations—Infirmaries and their Coal Supplies—The Monotony of Convalescence—Negro Patients at Bristol Royal Infirmary—Insanity and Economics—Sanatorium Advantages for Infirmary Consumptives—Poor-Law Medical Officers and the Cause of Reform — New Orthopædic Hospital for Wounded Soldiers—Hospital Chaplains and the Hospital Church—Christmas Festivities in Summer—Italy and the Drug Market.

**The Position of War Consultants** ... ... 309

The Government's New Policy.

**The Military Cross for Medical Heroism** ... 310

Decorations for Colonial Doctors.

**Doctors' Wills** ... ... ... ... ... ... 310

**The War and Dietaries in Poor-Law Institutions** ... ... ... ... ... ... 311

The Proper Way to Economise.

**Medical Appointments** ... ... ... ... 311

[*See over.*]

### CONTENTS—(*continued*).

| | PAGE |
|---|---|
| **Research Defence Society** ... ... ... ... ... | 312 |
| Sir William Osler, Bart., on Some Results of the War. | |
| **Gifts and Bequests** ... ... ... ... ... ... | 312 |
| **The Treatment of Wounds in War** ... ... | 313 |
| **Bravery in the Ranks** ... ... ... ... | 314 |
| The Work of the Overseas Medical Corps. | |
| **A Medical Causerie** ... ... ... ... ... | 315 |
| **Round the World** ... ... ... ... ... ... | 316 |
| Fellow-Workers and the Roll of Honour. | |
| **Hospital Architecture and Construction** ... | 317 |
| The New Chelsea Hospital for Women. | |
| **Latest Installations in Hospital Equipment** ... | vi |
| What Others are Doing. | |
| **The Editor's Letter-Box** ... ... ... ... | 320 |
| A List of Medical Offers of Service. | |
| Hospitals and the Spirit Tax: Mr. F. A. Hocking's Proposals. | |
| **The Institutional Worker** ... ... (Suppl.) | 1 |
| The Admission of In-Patients: Answer to June Question. | |
| Question for July. | |
| Questions Invited. | |
| Enquiries and Answers. | |
| Practical Matters: War Economy—The Value of Old Electric-Lamp Caps. | |
| Employment and Training: Territorial Nursing—Massage Training in the Colonies—Preliminary Training. | |

